# Comparison of the long-term efficacy of tenofovir and entecavir in nucleos(t)ide analogue-naïve HBeAg-positive patients with chronic hepatitis B: A large, multicentre, randomized controlled trials

**DOI:** 10.1097/MD.0000000000013983

**Published:** 2019-01-04

**Authors:** Dachuan Cai, Chen Pan, Weihua Yu, Shuangsuo Dang, Jia Li, Shanming Wu, Nan Jiang, Maorong Wang, Zhaohua Zhang, Feng Lin, Shaojie Xin, Yongfeng Yang, Baoshen Shen, Hong Ren

**Affiliations:** a The Second Affiliated Hospital of Chongqing Medical University, Chongqing; b Mengchao Hepatobiliary Hospital of Fujian Medical University, Fuzhou; c The Eighth People's Hospital of Guangzhou; d The Second Affiliated Hospital of Xi’an Jiaotong University, Xi’an; e The Second People's Hospital of Tianjin; f Shanghai Public Health Clinical Center Shanghai; g Sichuan Provincial People's Hospital, Chengdu; h No.81 Hospital of PLA; i Jinan Infectious Disease Hospital; j Hainan General Hospital, Haikou; k No.302 Hospital of PLA, Beijing; l The Second Hospital of Nanjing, Nanjing; m The First Affiliated Hospital of Xinxiang Medical University, Xinxiang, China.

**Keywords:** efficacy, entecavir capsule, safety, tenofovir disoproxil fumarate capsule

## Abstract

**Objective:**

We conducted this study to compare the efficacy and safety of entecavir and tenofovir in the treatment of treatment-naïve HBV e antigen (HBeAg)-positive patients with chronic hepatitis B (CHB) for 144 weeks.

**Methods:**

A total of 320 treatment-naïve HBeAg-positive CHB patients who received randomly a single regimen of either entecavir capsule (ETV) (n = 160) or tenofovir disoproxil fumarate capsule (TDF) (n = 160) for 144 weeks were consecutively enrolled from 14 tertiary hospitals or university hospitals in China between January 2012 and December 2014.

**Results:**

Two groups showed no difference in baseline characteristics. After 144 weeks of treatment, HBV DNA levels were similarly suppressed in both groups (ETV vs TDF; -6.6485 vs −6.692 log10IU/mL, *P* = .807). At the same time, both groups showed no difference in terms of the serologic and biochemical response. Of all patients, 2 dropped out due to adverse events and 5 experienced serious adverse reactions.

**Conclusion:**

Both capsules (ETV or TDF) were equally effective in nucleos(t)ide-naive CHB patients with a comparable side-effect profile even in a long-term of 144 weeks.

## 1 Introduction

Hepatitis B virus (HBV) infection is a severe health problem, which affected more than 400 million people worldwide. After its chronic infection, about 25% people may develop end-stage liver disease (ESLD), cirrhosis, liver failure, or hepatocellular carcinoma (HCC).^[1]^ Effective antiviral treatment can prevent liver fibrosis, decompensated cirrhosis, and hepatocellular carcinoma incidence by decreasing HBV DNA levels and ameliorating liver inflammation.^[2,3,4,5]^

At present, according to several international guidelines, entecavir (ETV) and tenofovir disoproxil fumarate (TDF) have a higher antiviral potency and higher genetic barriers. As a result, both are recommended as the first-line therapy in the treatment -naïve HBV e antigen (HBeAg)-positive adults with chronic hepatitis B virus (CHB) infection.^[6–8]^ Although there have been some reports comparing the short-term efficacy directly between TDF and ETV in treatment-naïve CHB, but there are no randomized controlled or well matched comparative studies regarding the efficacy of TDF or ETV in treatment-naïve CHB patients with a longer period of treatment. For example, the follow-up time of those studies were no more than 72 weeks (most of them were 48 weeks), and observed population is small.^[9–13]^ Therefore, we performed a large, multicentre, randomized controlled trials (RCT) study to directly compare the efficacy and safety of TDF and ETV in nucleos(t)ide analogue (NA) -naïve HBeAg-positive patients with CHB with a treatment period of 144 weeks.

## 2 Methods

### 2.1 Patients

A total of 320 treatment-naïve HBeAg-positive CHB patients who received a single regimen of either ETV (n = 160) or tenofovir disoproxil fumarate capsule (TDF) (n = 160) for 144 weeks were consecutively enrolled from 14 tertiary hospitals or university hospitals in China between May 2012 and December 2015, where the full trial protocol can be accessed.

The diagnosis of CHB was based on the AASLD, the APASL, and the CSH/CSID, CMA guidelines.^[6–8]^ Adult patients (60 ≥ age ≥ 18) with a strict diagnosis of CHB were eligible for inclusion if they were without the following exclusion criteria:

1. coinfection with other kinds of hepatitis virus (like hepatitis C virus) or the human immunodeficiency virus, or suffered from co-morbidities with alcoholic, drug-induced or autoimmune liver diseases;
2. with a previous history of NA or interferon-alfa therapy;
3. with evidences of liver cirrhosis or HCC;
4. with a history of solid organ transplantation;
5. pregnant or lactating women.

All patients gave their informed consent before their inclusion in the study. This study was approved by the Ethics Committee of the second affiliated hospital of Chongqing Medical University and other hospitals in accordance with the principles stated in the Declaration of Helsinki.

### 2.2 Study design and drugs

The 2 phases of the multicentre prospective trial are double-blind and double-mimic randomized control trial for 48 weeks and open trial for another 96 weeks. The experiment is based on the stratified random method. The random assignment code was simulated by statisticians using the SAS software. All random grouping numbers were sent to the clinical trial center and equipped with corresponding medicine boxes. The patient was treated according to the order of the visit and the medicine boxes with the same serial number. Both the patients and the investigators were blinded after assignment to treatment at the first stage (48 weeks). Because the appearance of test drug (TDF capsule) and control drug (ETV capsule) was so different than the simulation method was applied during the trial. For those 360 patients, TDF or ETV use was randomly assigned at a ratio of 1:1. All the drugs, either TDF capsule or ETV capsule, were provided by Cosunter Pharmaceutical (Ningde, Fujian, China).

### 2.3 Serum assay and methodology

All serum samples were assessed in each local clinical center laboratory with a standard procedure. Serum HBV surface antigen (HBsAg), HBV surface antibody (HBsAb), HBeAg, HBV e antibody (HBeAb), and HBV core antibody (HBcAb) were measured using microparticle enzyme immunoassay (Abbott Architect, North Chicago, IL). Serum HBV DNA levels were measured using a COBAS TaqMan PCR assay with a lower limit of detection of 20 IU/mL (Roche, Branchburg, NJ). The calculation of FIB-4, APRI, and eGFR were based on the literature.^[22,23,24]^

### 2.4 Statistical analysis

Study treatments were compared for noninferiority. Sample size was determined on the basis of the primary analysis of the number of viral load drops (Logarithmic form). According to the references, the number of viral load drops in the treatment of CHB with the control drug (ETV) decreased to −6.89, SE = 0.108, N = 354, and the standard deviation was 2.03. Based on the clinical significance and statistical principle, the non-inferior-effect margin was set to -0.7. The parameters used are: α = 0.025, β = 0.2, δ = −0.7, which meant that there should be 133 patients in each group. Assuming a dropout rate of 20% during a 144 weeks period, the number of patients required for each group was estimated to be 160. As a result, the total number of individuals was 320, which was twice that of the number given in each group. Statistical analyses in this study were performed using Statistical Package for the Social Sciences software, version 20.0 (SPSS 20.0, IBM, Armonk, NY). Quantitative variables were calculated by the Student *t* test, or Mann–Whitney *U* test when appropriate, with a presentation of median (range) or mean ± standard deviation (SD). Categorical variables were analyzed as proportions (%) and compared using Pearson *χ*2 test. Statistically significant difference was considered when *P*-values < .05. And all tests for significance were 2-tailed.

## 3 Results

### 3.1 Baseline characteristics of enrolled patients

This study comprised of 2 groups, with a total of 315 patients with CHB who were qualified for FAS analysis at the end of the trial, 157 in TDF group and 158 in ETV group, respectively. Some data at baseline such as gender, age, ethnics, weight, height, and HBV DNA level, showed no significant difference between the TDF group and ETV group (Table 1).

**Table 1:**
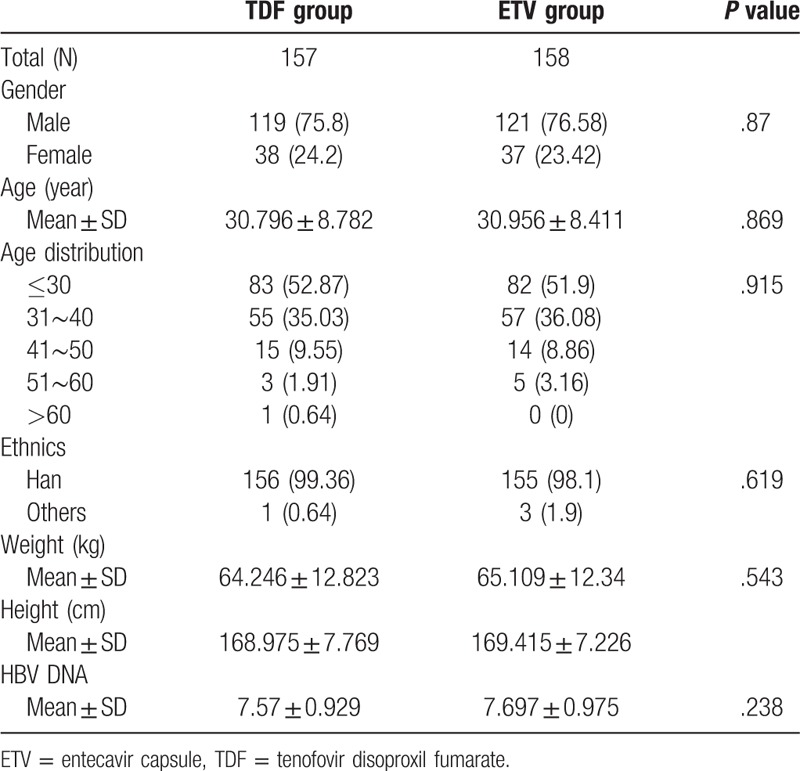
Baseline characteristics of patients recruited in the study.

### 3.2 Compare the liver biochemical response of CHB patients in TDF group versus ETV group

**Figure 1:**
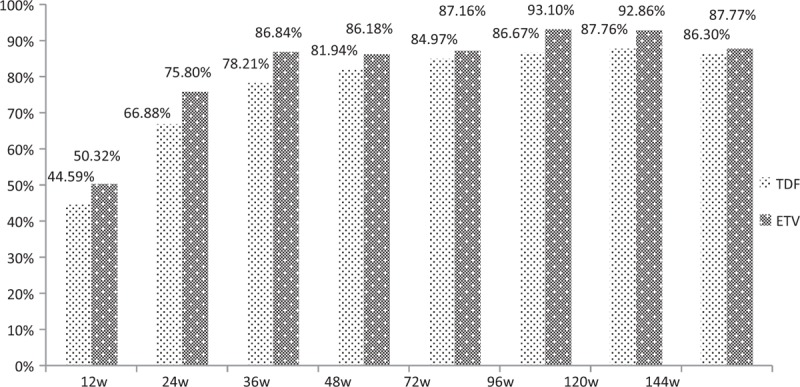
The incidence of ALT normalization in TDF and ETV group at each observational point. ALT = alanine aminotransferase, ETV = entecavir capsule, TDF = tenofovir disoproxil fumarate.

Normalization of liver biochemical index is the basic target of antiviral response. During a total of 144 weeks follow-up, the incidence of alanine transaminase (ALT) normalization in TDF and ETV group was 86.30% and 87.77%, respectively (*P* = .712). And at each observational point, the incidence of ALT normalization in TDF and ETV group showed almost no significant difference (at week 4: 10.19% vs 16.26%, *P* = .102; at 12 weeks: 44.59% vs 50.32%, *P* = .309; at 24 weeks: 66.88% vs 75.80%, *P* = .081; at 48 weeks: 81.94% vs 86.18%, *P* = .31; at 72 weeks: 84.97% vs 87.16%, *P* = .583; at 96 week: 86.67% vs 93.1%, *P* = .067; at 120 weeks: 87.76% vs 92.86%, *P* = .145), except at week 36 (78.21% vs 86.84%, *P* = .046) (Fig. 1).

### 3.3 Compare the viral response in CHB patients in TDF group versus ETV group

**Figure 2:**
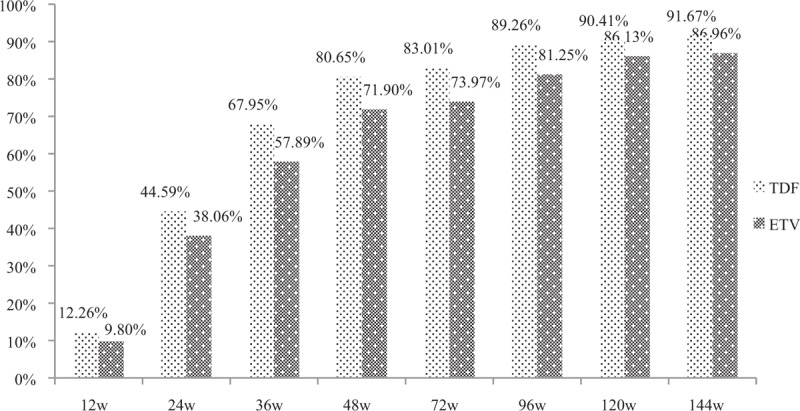
The incidence of undetectable HBV DNA in TDF and ETV group at each observational point. ETV = entecavir capsule, HBV = hepatitis B virus, TDF = tenofovir disoproxil fumarate.

Viral response is an important target of antiviral therapy. In this study, we found that the incidence of undetectable HBV DNA in TDF group and ETV group were not significantly different at each observational point (at 12 weeks: 12.26% vs 9.8%, *P* = .492; at 24 weeks: 44.59% vs 38.06%, *P* = .242; at 36 weeks: 67.95% vs 57.89%, *P* = .068; at 48 weeks: 80.65% vs 71.9%, *P* = .071; at 72 weeks: 83.01% vs 73.97%, *P* = .057; at 96 week: 89.26% vs 81.25%, *P* = .053; at 120 weeks: 90.41% vs 86.13%, *P* = .262; at week 144: 91.67% vs 86.96%, *P* = .2) (Fig. 2).

### 3.4 Compare the HBeAg loss/seroconversion of CHB patients in TDF group versus ETV group

**Figure 3:**
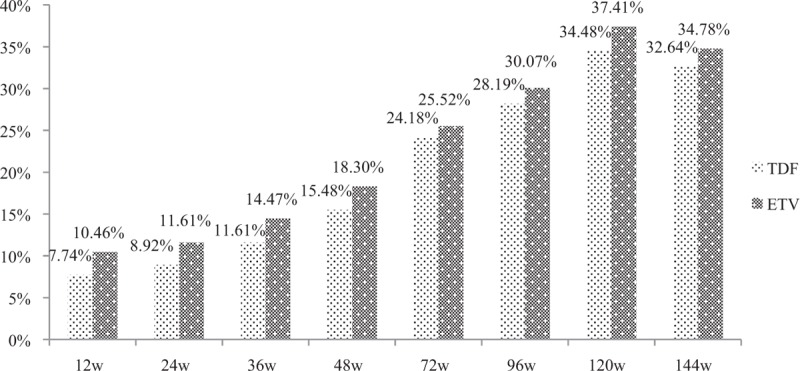
The incidence of HBeAg loss in TDF and ETV group at each observational point. ETV = entecavir capsule, HBeAg = hepatitis B e antigen, TDF = tenofovir disoproxil fumarate.

**Figure 4:**
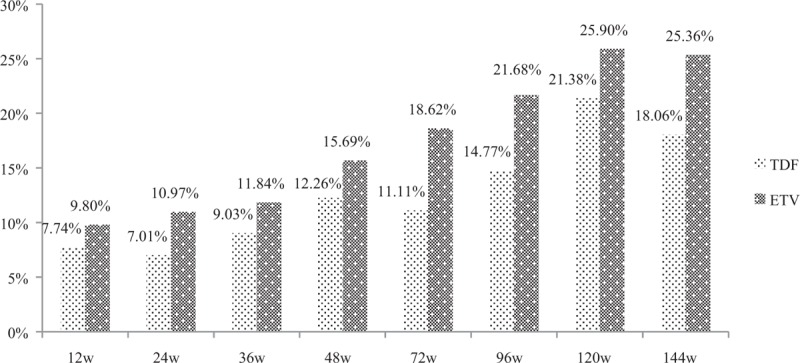
The incidence of HBeAg seroconversion in TDF and ETV group at each observational point. ETV = entecavir capsule, HBeAg = hepatitis B e antigen, TDF = tenofovir disoproxil fumarate.

HBeAg loss/seroconversion is a harder target of antiviral therapy. First of all, the incidence of HBeAg loss was observed. No significant difference of the HBeAg loss incidence was found at each observational point between the TDF group and the ETV group (at 12 weeks: 7.74% vs 10.46%, *P* = .407; at 24 weeks: 8.92% vs 11.61%, *P* = .433; at 36 weeks: 11.61% vs 14.47%, *P* = .457; at 48 weeks: 15.48% vs 18.3%, *P* = .509; at 72 weeks: 24.18% vs 25.52%, *P* = .79; at 96 week: 28.19% vs 30.07%, *P* = .723; at 120 weeks: 34.48% vs 37.41%, *P* = .607; at week 144: 32.64% vs 34.78%, *P* = .703) (Fig. 3). Similar results were obtained in consideration of the incidence of HBeAb seroconversion: there were no significant differences of the HBeAg seroconversion incidence between the TDF group and ETV group at each observational point (at 12 weeks: 7.74% vs 9.8%, *P* = .522; at 24 weeks: 7.01% vs 10.97%, *P* = .221; at 36 weeks: 9.03% vs 11.84%, *P* = .42; at 48 weeks: 12.26% vs 15.69%, *P* = .385; at 72 weeks: 11.11% vs 18.62%, *P* = .068; at 96 week: 14.77% vs 21.68%, *P* = .125; at 120 weeks: 21.38% vs 25.9%, *P* = .37; at week 144: 18.06% vs 25.36%, *P* = .136) (Fig. 4).

### 3.5 Compare the combined response of CHB patients in TDF group versus ETV group

**Figure 5:**
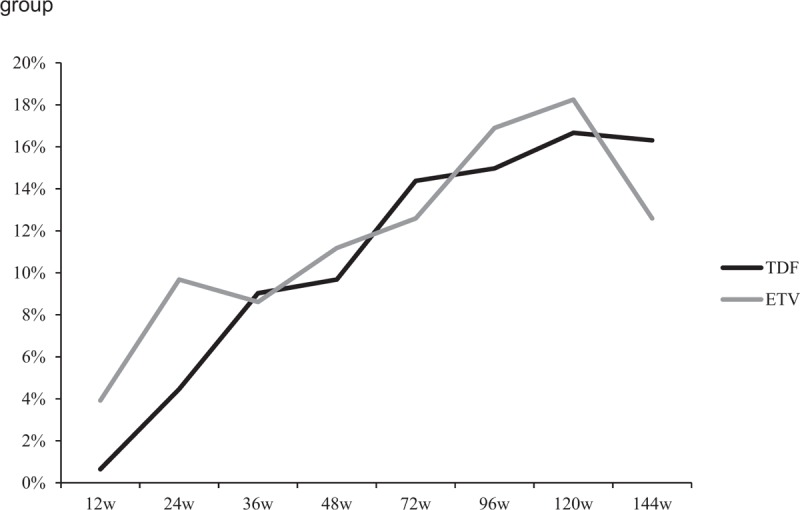
Compare the combined response of CHB patients in TDF group versus ETV group. CHB, chronic hepatitis B, ETV = entecavir capsule, TDF = tenofovir disoproxil fumarate.

At the end of the double-blind trial (after 48 weeks), the percentage of subjects who reached the joint response (The joint response was defined as one with undetectable or lower than the detection limit HBV DNA (PCR) and negative HBeAg and normalized ALT level.) was 9.68% and 11.18%, respectively, with no statistically significant difference between the groups (*P* = .666). After 96 weeks of open trial, the percentage of subjects who reached the joint response was 16.31% and 12.59%, respectively, and the difference between the groups was not statistically significant (*P* = .38). There was no statistically significant difference between the 2 groups in the percentage of subjects in the joint response (*P* > 0.05) (Fig. 5).

### 3.6 Progression of disease

**Figure 6:**
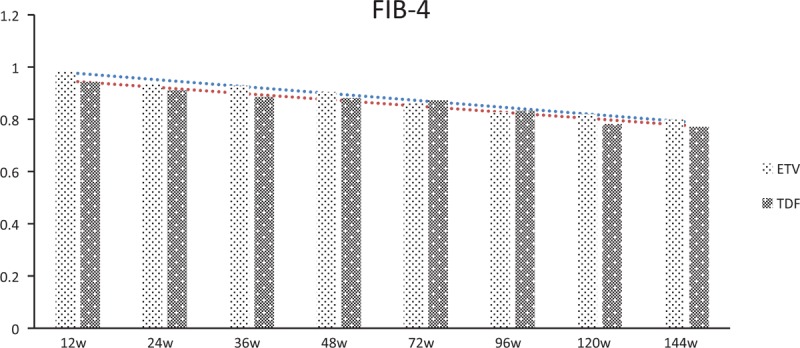
Fluctuation of the Fib-4 scoring of CHB patients in TDF group versus ETV group. CHB, chronic hepatitis B, ETV = entecavir capsule, TDF = tenofovir disoproxil fumarate.

**Figure 7:**
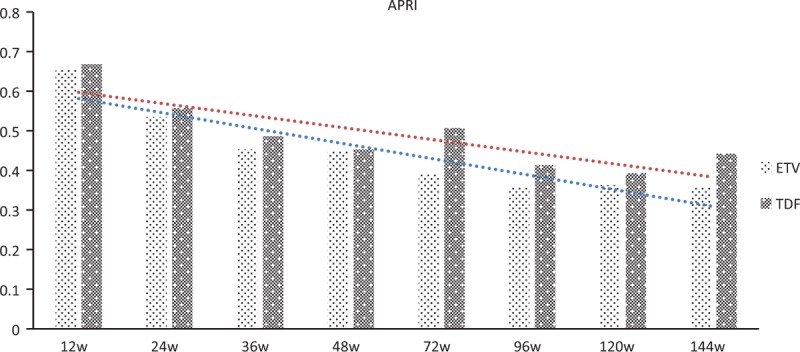
Fluctuation of the APRI scoring of CHB patients in TDF group versus ETV group. CHB, chronic hepatitis B, ETV = entecavir capsule, TDF = tenofovir disoproxil fumarate.

During the observational period, there was no incidence of hepatic decompensation, hepatocellular carcinoma, or cirrhosis. The tendencies of regression of fibrosis have been observed through Fib-4 and APRI scoring, with no statistically significant difference between ETV and TDF groups (*P* = .486 and *P* = .371 respectively) (Figs. 6 and 7).

### 3.7 Primary treatment failure

Primary treatment failure was defined as reduction of serum HBV DNA by < 2log10IU/mL at week 24 of antiviral therapy in an adherent patient. There were 4 cases (2.55%) in the TDF group and 1 (0.65%) in the ETV group with primary treatment failure respectively. There was no statistically significant difference between the groups (*P* = .375).

### 3.8 Virological and biochemical breakthrough

There were 5 cases (3.18%) and 6 cases (3.8%) with virological breakthrough in the TDF group and the ETV group respectively till week 144. The difference between the 2 groups was not statistically significant (*P* = .767). There were 3 cases (1.91%) and 1 (0.63%) with biochemical breakthrough in the TDF group and the ETV group, respectively. There was no statistically significant difference between the 2 groups (*P* = .61).

### 3.9 Safety data

**Figure 8:**
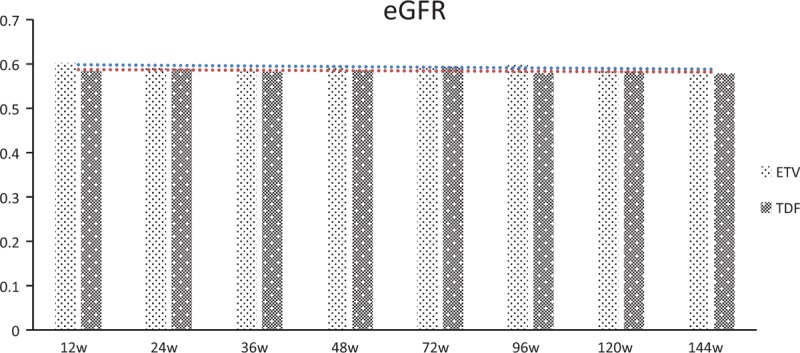
Fluctuation of the eGFR of CHB patients in TDF group versus ETV group. CHB, chronic hepatitis B, ETV = entecavir capsule, TDF = tenofovir disoproxil fumarate.

There were 320 cases in SS (safety set) analysis, 160 in each group. The incidence of adverse events in the TDF group was similar to that of the ETV group, with 45% and 48.13% (*P* > .05), respectively, and the incidence of adverse effects was 31.25% and 35% (*P* > .05). Both of those 2 drugs were generally well tolerated. Common adverse effects were elevated creatine kinase, lactate, alkaline phosphatase, or alanine aminotransferase, total bilirubin, white cells, blood sugar, and depleted white cells, red blood cells or hemoglobin. Other mild adverse effects were fatty liver or headache. Renal function should be carefully monitored during treatment with NAs. The comparison for the change of estimated glomerular filtration rate (eGFR) by MDRD showed that there was no obvious fluctuation of the eGFR during the observational time even though there was a *P* value less than .007 between the TDF group and ETV group (Fig. 8).

## 4 Discussion

The goals of anti-HBV treatment are to improve quality of life and survival of the infected person by maximally suppressing HBV replication, reducing hepatic necroinflammation and hepatic fibrosis, and delaying and decreasing hepatic failure, progression of hepatic decompensation, HCC, and other complications. Thus, the sustained suppression of HBV replication should be considered the most important therapeutic goal for CHB patients in clinical settings. Currently, ETV and TDF are the most commonly used HBV drugs due to their excellent potency and higher genetic barrier. As a result, both ETV and TDF were recommended as first-line therapy for CHB by the international guidelines.^[6–8]^ However, there is limited information on the comparative efficacy of these drugs. In fact, TDF and ETV are relatively new antiviral NAs with proved effect in suppressing HBV DNA replications with minimal drug resistance,^[14–16]^ which can effectively reduce the risk of HBV-related ESLD.

As reported, approximately 81% CHB patient obtained a sustained viral response (SVR) after receiving 3 years of TDF therapy.^[17]^ And ETV can effectively suppress the serum HBV DNA levels in more than 75% patients after 48 weeks of treatment.^[18]^ Several studies have compared the efficacy of TDF and ETV directly. In a follow-up study of 94 patients, Doğan ÜB et al found that the virologic response and tolerability did not significantly differ between these 2 kinds of drugs in the first year of treatment.^[9]^ Similar results were confirmed in other studies with a larger population (n = 345),^[10]^ or a longer follow-up time (72 weeks).^[12]^ However, in general, studies which directly compared the efficacy of TDF and ETV are lack, and the follow-up time is not long enough.

In this study, a large, multicentre, RCT cohort was performed, to compare the efficacy of TDF and ETV in NA-naïve patients with CHB. We found that after receiving the antiviral treatment of TDF or ETV, the incidence of liver biochemical response, viral response, and HBeAg loss/seroconversion were all not significantly different (all *P* > .05). Compared to interferon that enhances the host immune system to mount defense against HBV, oral NA therapy (including TDF and ETV) confers low HBeAg seroconversion rates. In this study, the rates of HBeAg loss or seroconversion seemed lower than the clinical trials or real-world reports in the Asian–Pacific region. For example, Ke et al^[19]^ pointed out that pooled HBeAg seroconversion for TDF was similar to the ETV group (24 weeks: 28% vs 29%, RR = 0.86, 95% CI = 0.45–1.66; 48 weeks: 16% vs 10%, RR = 1.09, 95%CI = 0.57–2.11) in a meta-analysis. Generally speaking, TDF and ETV do not greatly influence HBeAg seroconversion for HBV patients. Therefore, it is plausible that TDF and ETV may have yielded lower rates of seroconversion with a marginal host immune system impact for different clinical trial population. As a clinical trial with a longer observation time of 144 weeks, regression of fibrosis has been observed with FIB-4 and APRI, which was coincident with previous results.^[9–13]^ With a much longer follow-up time (144 weeks) and a larger population (n = 315), this study filled a gap in this field to some extent. In the present study, there were no statistically significant differences between tenofovir and ETV in HBV DNA suppression to undetectable levels till week 144. When comparing the response rates overall in the patients, our results can be interpreted as ETV and tenofovir treatment being equally effective in CHB patients.

Multi-center, long-term studies using ETV and/or TDF have shown excellent safety and tolerance with little antiviral resistance (1.2% in ETV and 0% in TDF up to 5 years). In our study, there was no serious clinical adverse reaction in those 2 treatment groups. On week 48, there were 2 cases of virological breakthrough in the TDF group; however, sequencing had showed that there was no known possible TDF-related mutation for 1 patient. It is known through query that the patient did not take the medicine as prescribed due to personal reason. Furthermore, the level of HBV DNA went down to undetected level again after the patient took TDF as prescribed for another 48 weeks (Table 2). We did not observe ETV-related resistance in this study, which may be due to the short observation period and the limited number of patients in the study. Patient adherence may contribute to achieve comparable overall virological response during long-term treatment period in both groups. For the safety reason, it would be necessary for the comparison for the change of estimated glomerular filtration rate (eGFR) for patients under NAs treatment, especially TDF. The comparison for the change of estimated glomerular filtration rate (eGFR) by MDRD showed that there was no obvious fluctuation of the eGFR during the whole observational time and there is almost no disparity between the TDF group and ETV group even though there was a *P* value less than .007. It was also coincident with previous results that patients who received tenofovir were no more likely to have changes in renal function than patients treated with ETV.^[20]^ The reason should be strict scrutinization of those patients at the baseline. The general low level of eGFR should be due to the patients population of Chinese.^[21]^

**Table 2:**
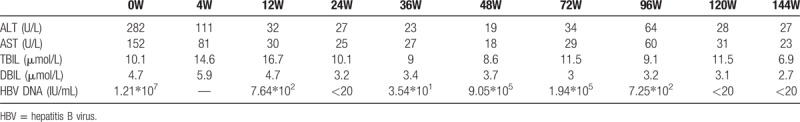
Evolution of virological and biochemical index during the virological breakthrough and re-treatment for one case.

There are some limitations of this study. First of all, there is no demonstration of a histological activity improvement at the observation point. Second, Genotypes were not analyzed; however, genotype B and C are the predominant genotypes in China, and genotype is not normally determined for naive CHB patients treated with NAs. Third, not all patients with virologic breakthroughs were sequenced for possible resistance-related mutation.

In conclusion, ETV and TDF were equally effective in nucleos(t)ide-naive CHB patients with a comparable side-effect profile even in a long-term of 144 weeks.

## Author contributions

**Data curation:** Dachuan Cai, Chen Pan, Weihua Yu, Shuangsuo Dang, Jia Li, Shanming Wu, Nan Jiang, Maorong Wang, Zhaohua Zhang, Feng Lin, Shaojie Xin, Yongfeng Yang, Baoshen Shen, and Hong Ren.

**Formal analysis:** Dachuan Cai and Hong Ren.

**Funding acquisition:** Hong Ren.

**Investigation:** Dachuan Cai, Chen Pan, Weihua Yu, Shuangsuo Dang, Jia Li, Shanming Wu, Nan Jiang, Maorong Wang, Zhaohua Zhang, Feng Lin, Shaojie Xin, Yongfeng Yang, Baoshen Shen, and Hong Ren.

**Project administration:** Dachuan Cai, Chen Pan, Weihua Yu, Shuangsuo Dang, Jia Li, Shanming Wu, Nan Jiang, Maorong Wang, Zhaohua Zhang, Feng Lin, Shaojie Xin, Yongfeng Yang, Baoshen Shen, and Hong Ren.

**Supervision:** Hong Ren.

**Writing – original draft:** Dachuan Cai.

**Writing – review & editing:** Dachuan Cai and Hong Ren.
